# Microsites Matter: Improving the Success of Rare Species Reintroductions

**DOI:** 10.1371/journal.pone.0150417

**Published:** 2016-03-01

**Authors:** Peter W. Dunwiddie, R. Adam Martin

**Affiliations:** Center for Natural Lands Management, Olympia, Washington, United States of America; University of Saskatchewan, CANADA

## Abstract

Our study was undertaken to better understand how to increase the success rates of recovery plantings of a rare hemiparasite, golden paintbrush (*Castilleja levisecta—*Orobanchaceae). This species is endemic to western Washington and Oregon, USA, and southwestern British Columbia, Canada. Over 5000 golden paintbrush plants were outplanted as plugs in 2007 at six different native prairie sites that were considered to be suitable habitat, based on general evaluations of vegetation and soil conditions. Outplantings were installed at regular intervals along transects up to 1 km long to include a range of conditions occurring at each site. All plantings were re-examined five years later. The patchy distribution of surviving plugs and new recruits within each reintroduction site suggested success is strongly influenced by microsite characteristics. Indicator species analysis of taxa growing in microsites around outplanted golden paintbrush identified species that were positively or negatively associated with paintbrush survival. Species such as *Festuca roemeri*, *Eriophyllum lanatum*, and *Viola adunca* were strong indicators at some sites; non-natives such as *Hypochaeris radicata* and *Teesdalia nudicaulis* tended to be frequent negative indicators. Overall, higher richness of native perennial forbs was strongly correlated with both survival and flowering of golden paintbrush, a pattern that may reflect interactions of this hemiparasite with the immediately surrounding plant community. Topographic position also influenced outcomes, with greater survival occurring on mounds and in swales, where soils generally were deeper. Our findings suggest that assessments of site suitability based on vegetation alone, and coarser, site-level assessments that do not characterize heterogeneity at the microsite scale, may not be strong predictors of restoration success over the longer term and in sites with variability in vegetation and soils. By identifying suitable microsites to focus rare species plantings, survival and efficiency may be significantly enhanced.

## Introduction

The restoration of rare species is a central focus of many conservation efforts. To reduce the likelihood of extirpation or extinction, such efforts generally focus on a variety of strategies to minimize threats and enhance the viability of populations. Actions often are directed towards increasing the size of precariously small extant populations (augmentation), establishing new populations that may increase a species’ range or distribution (translocation, introduction, or reintroduction), improving the suitability of sites to sustain populations by removing competitors and predators (habitat enhancement), and restoring key ecological processes such as fire, pollinators, or keystone species and ecosystem engineers [1–3]. However, deriving generalizable conclusions from reintroduction outplantings is difficult because they often are not designed in ways that allow rigorous analyses of results, they may be monitored for only a year or two, involve few individuals, or may occur at only one or two sites [4]. Thus, the factors contributing to or impeding success are often poorly understood [4,5], and longer-term outcomes, and even the ultimate success or failure of restoration plantings, may be unknown [6].

Authors have increasingly advocated that restoration plantings be carried out more as scientific experiments, with explicit hypotheses, replication within and between sites, and careful tracking of planting success over several years [7,8]. Such an approach is especially important in light of Godefroid et al.’s [4] meta-analyses of rare plant reintroduction efforts, in which they found that the reasons reported for reintroduction failure fell largely into two categories: “unknown” (34%) and “unsuitable habitat” (29%). Clearly, more rigorously designed and monitored restoration plantings are needed to better understand the factors that contribute to success or failure, and reduce the size of the “unknown” category. However, an experimental approach is also critical for clarifying what constitutes suitable habitat for a rare species. Rare plant introductions can occur in “unsuitable habitat” for reasons that may not be solved simply by careful matching of native habitat with the ecology of receptor sites [7]. For example, sites appearing well-suited for reintroduction may not exist, and restorationists may be forced to use sub-optimal sites. But with many rare species, understanding the myriad aspects of soils, vegetation, genetics, disturbance, and environment that collectively constitute suitable habitat for sustaining viable populations is woefully inadequate [7]. In such cases, a direct experimental approach that rigorously evaluates sites using outplanted individuals may be essential to enhance the success of reintroductions.

In our study, we adopted an experimental approach to identify suitable habitat for establishing new populations of a federally threatened species, golden paintbrush (*Castilleja levisecta—*Orobanchaceae), in sites where it had not previously been known to exist. We were particularly interested in understanding why previous efforts to establish this species at sites that had been deemed most similar to those with extant golden paintbrush populations [9] often produced widely variable results, even when seed or plugs were planted within a few meters of one another (Dunwiddie, personal observation). We hypothesized that such patterns might result from variability in habitat suitability on a microsite scale, and that merely matching the characteristics of occupied sites with potential receptor sites was insufficient to ensure success of outplantings. By identifying features of microsites associated with longer-term survival of experimentally outplanted golden paintbrush, we sought to gather information that could help increase the success rates of other recovery plantings by allowing practitioners to identify suitable planting locations more precisely, and lead to better establishment of viable recovery populations.

### Golden Paintbrush

Historically, golden paintbrush occurred in low elevation prairies in western Oregon, Washington, and southwestern British Columbia [10]. These prairies have been reduced to a fraction of their original extent due to conversion to agriculture, residential development, and encroachment of Douglas-fir forest [11]. The native flora of the remaining prairies has also declined with the cessation of Native American burning and the extensive invasion of species such as Scotch broom (*Cytisus scoparius*), tall oatgrass (*Arrhenatherum elatius*), and hairy cat’s-ear (*Hypochaeris radicata*). By the time golden paintbrush was listed as threatened in the U.S. in 1997, it had been extirpated from Oregon, and only persisted in a dozen sites [10]. A federal Recovery Plan and funding from the U.S. Fish and Wildlife Service attracted a considerable amount of research on the biology and ecology of the species [9,12–25].

Like many other members in the Orobanchaceae, golden paintbrush is a generalist hemiparasite, capable of attaching itself to the roots of other species, including grasses, forbs, and shrubs. The species is of particular interest not only because it is rare; it also is a larval host for the recently listed, federally endangered butterfly, Taylor’s checkerspot (*Euphydryas editha taylori*) [25]. The few natural populations of paintbrush that still exist occur mostly in very small prairie remnants, and on bluffs and rocky balds close to the coast in northern Puget Sound [15]. In recent years, some of these populations have, at times, been reduced to fewer than 100 plants.

To remove golden paintbrush from its current “threatened” status, several criteria in the federal Recovery Plan must be met. The species must occur in at least 20 stable populations distributed throughout the historical range of the species. To be considered stable, the populations must maintain a 5-year running average of at least 1000 flowering individuals, and be sustained by natural regeneration. Of the 20 populations, at least 15 must be on protected sites where management of golden paintbrush is a primary objective [10]. To reach this goal, many existing populations must be augmented, new populations established, habitat quality enhanced, and threats abated. This study focuses on the South Puget Sound region in Washington, where extensive efforts are being made to enhance habitats and establish multiple new golden paintbrush populations.

## Methods

### Study Area and Design

**Fig 1:**
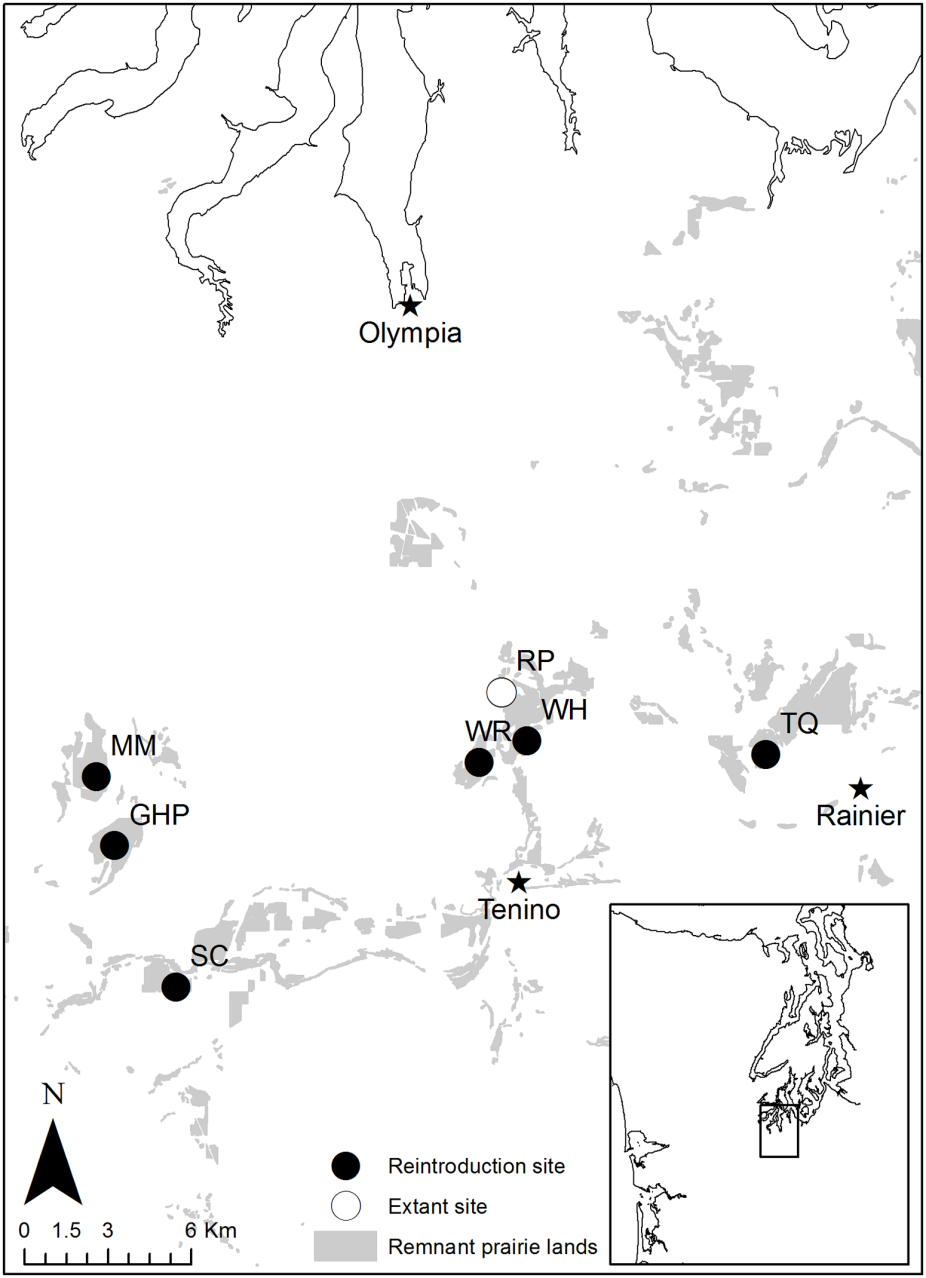
Location of study sites in the South Puget Sound region of western Washington.

Many of the largest and most intact native prairies in western Washington occur in South Puget Sound. Although only a single natural population of golden paintbrush persists in this region (Rocky Prairie, 46°55’11”N, 122°51’34”W, Fig 1), there are many sites being managed for conservation objectives, and the area is a very high priority for establishing new populations [9]. Experimental plugging and seeding of golden paintbrush were carried out at several South Sound sites beginning in 2002 by Dunwiddie and colleagues. However, these efforts were limited by small quantities of wild-collected seed and nursery-grown plugs, and little understanding of where the species would survive most successfully. As a result, few plants became established. Survival in these early plantings varied widely even within a single site, suggesting that microsite differences played an important role in seedling and plug performance.

To better understand which microsite characteristics are closely associated with golden paintbrush success, we set up a large-scale experimental outplanting of nursery-grown plugs at six native prairies in South Puget Sound in 2007 (Fig 1). Sites included Scatter Creek (SC—223ha, 46°49’23”N, 123°00’31” W; Washington Department of Fish and Wildlife), Mima Mounds (MM—150ha, 46°53’58”N, 123°03’10”W; Washington Department of Natural Resources), Glacial Heritage (GH—235ha, 46°52’04”N, 123°02’54”W; Thurston County), Wolf Haven (WH– 13ha, 46°54’13”N, 122°50’51”W; Wolf Haven International), West Rocky (WR—70ha, 46°53’52”N, 122°52’13”W; Washington Department of Fish and Wildlife), and Tenalquot (TQ—40ha, 46°54’02”N, 122°43’59”W; The Nature Conservancy). The first three are among the larger and most floristically-intact prairie remnants in the region; the last two are smaller and have a relatively low diversity of native species [26]. WH is the smallest of the sites, but is reasonably diverse for its size. Permission was granted by the respective landowning agencies and organizations to carry out this study on their properties; Washington State Parks, Whidbey-Camano Land Trust, the United States Department of Defense, and The Nature Conservancy granted permission to collect seed from wild golden paintbrush populations.

Soils at all sites are described either as Spanaway (gravelly sandy loam), Spanaway-Nisqually complex, or Nisqually (loamy fine sand) (http://websoilsurvey.nrcs.usda.gov/app/WebSoilSurvey.aspx). All developed on glacial outwash, and the abundance of soils mapped as Spanaway-Nisqually complex testifies to the frequency with which they intergrade with one another. The distinctive mounded topography widely known as “Mima mounds” is especially prevalent in this region, and is predominant at all sites except TQ and SC. Such areas are mapped as Spanaway-Nisqually complex, with the deeper, finer Nisqually soils typically comprising the mounds, and the coarse, gravelly or cobbly Spanaway soils occupying the intermounds. TQ is flat, and consists entirely of coarse Spanaway soils. Nisqually soils are only mapped in portions of SC and WR, but Rocky Prairie (mapped as Spanaway-Nisqually complex) has sizeable areas with predominantly Nisqually characteristics. Golden paintbrush grows especially well in the relatively deep, rich Nisqually loams [23], and we have suggested [25] that this species historically may have been especially prevalent in these soils before they were converted to agriculture.

Golden paintbrush plants were grown in the nursery in 106 cm^3^ conical tubes for one year using seed collected from four different wild populations, and then outplanted into the prairies in November, 2007. Five plugs—one derived from each source population, with one repeat—were planted systematically within 1 m^2^ quadrats to allow relocation of individuals without having to mark every plant. Pairs of quadrats were placed at 10m intervals (or at SC, 5m) along single or multiple transects that collectively extended nearly a kilometer across each prairie, and included at least 900 plants. Outplantings were monitored annually for five years. Each plant was recorded as dead or alive, and living plants were recorded as vegetative or flowering. In addition to tracking each outplanted plug, we also recorded any new golden paintbrush recruits that appeared in or near the quadrats each year.

Management within the six sites where the golden paintbrush were outplanted included application of herbicides to control invasive weeds and prescribed burning. These activities were carried out in various portions of some sites in different years, but were not systematically applied within the transects and across the sites in ways that would allow us to rigorously examine the effects of these treatments.

Many conditions may have affected the performance of the outplanted plugs. We were particularly interested in identifying microsite characteristics that might be readily discernable when sowing seed or planting plugs. As a hemiparasite, it seemed likely that species growing in close proximity to the golden paintbrush plugs might play a significant role in their growth and survival. Furthermore, small-scale variability in soil texture, depth, etc. likely corresponds with topographic position (mound, swale, level). Therefore, in 2012, we recorded all species present in each quadrat, as well as noting topographic position. For comparison with microsite conditions in an extant, nearby golden paintbrush population, we used species composition data from 422, 1 m^2^ quadrats at Rocky Prairie. Species nomenclature follows the Integrated Taxonomic Information System (http://www.itis.gov).

### Data Analysis

#### Indicator Species Analysis (ISA)

We used a modified version [27] of Indicator Species Analysis [28] to explore how species composition and prairie topography were related to golden paintbrush survival. Analyses were done separately for each of the six prairies in which outplantings occurred (GH, MM, TQ, WH, SC, and WR); in addition, we conducted a similar analysis using data from Rocky Prairie, where golden paintbrush grows naturally, to compare the species composition in quadrats where golden paintbrush was extant with locations where it did not occur. In the prairies where each plot was planted with golden paintbrush, inferences of negative indicators are particularly strong, since zero values are more likely due to factors within the quadrat, and not because the species had not yet colonized the location.

We conducted two types of ISA on data from plant communities associated with golden paintbrush, beginning with a conventional ISA for each site using the species presence matrix to determine which individual species were positive or negative indicators. Species were considered to be indicators if indicator values were ≥25 and p<0.05. Second, we examined functional group richness in relation to golden paintbrush performance. Functional groups were defined by life form (graminoid, forb, shrub), life history (annual, perennial), and origin (native, exotic). Functional groups were used so that broader generalizations might be made with other sites that include somewhat different species assemblages.

After values for each of the analyses were calculated, we used a meta-analytic approach to calculate averages across the sites. For each functional group, ISA values were weighted by sample size, and averaged at all sites where present. Next, *p* values associated with each functional group and topographic position were converted to Z-scores and averaged across each site, weighted by sample size. This averaged Z-score was then converted back into a *p* value (see [27], appendix B).

#### Site and Topography

We used a generalized linear model to determine how golden paintbrush survival is influenced by the interaction of site and topography. Both site and topography were treated as fixed effects because the sites in our study are still the subject to ongoing reintroduction efforts for the species, and their identity is important to local managers and practitioners. We tested the interaction of site and topography because we suspected the site histories and differences in soils between sites likely also change the impact of different topographic features on prairie plant communities and thus golden paintbrush survival. SC and TQ were excluded from this analysis because planting locations at both sites were in level terrain. We used the binomial glm function in the lme4 package [29] to run the model, and the ghlt function in the multcomp r package [30] to run post-hoc analysis using Tukey’s all pairs comparison. We used the effects package [31] to generate model average estimates and 95% confidence intervals.

#### Ecological Similarity

A previous study by Lawrence and Kaye [32] of golden paintbrush found the ecological similarity between extant and reintroduction sites (primarily in Oregon) was important at the site level. We ran the same analysis at the microsite level to determine whether the microsites in reintroduction sites where outplanted golden paintbrush survived were more similar to microsites in Rocky Prairie where golden paintbrush plants existed in a natural population. Rocky Prairie was chosen for comparison as it is the only extant golden paintbrush site in the South Puget Sound Region. We used a Bray-Curtis distance measure on the functional group matrix to calculate the average similarity between successful outplanted microsites and Rocky Prairie. For this analysis, the average richness for each functional group at Rocky Prairie was used to calculate similarity to all quadrats. We then ran a two-way ANOVA with a Tukey’s post-hoc test comparing similarity among sites and quadrats where golden paintbrush survived or died, as well as the interaction between site and paintbrush persistence. Lastly, we used regression analysis to assess how native perennial richness impacted golden paintbrush survival, hypothesizing that survival should increase as native perennial richness increased. To test this, we calculated the proportion of surviving plugs for each native perennial richness value (0,1,2….etc.) across all reintroduction sites. Thus *x* is the native perennial richness per quadrat, and *y* is the proportion of plants surviving across quadrats with that given native perennial richness. All analyses were done in R (version 3.1.1).

## Results

Golden paintbrush survival and vigor (as measured by the percent of outplanted plugs that flowered) for each site provide a general comparison of how the sites rank with one another. SC and MM were the top performers for both measures, WH ranked last in survival and second from last in flowering, and the other three sites were generally intermediate, depending on which measure of success was used (Table 1). Overall, an average of 84% (72–91%) of the plants at each site remained alive after Year 1; by Year 5, this average had dropped to only 15% (7–25%).

**Table 1:** Survival and Flowering of Outplanted Golden Paintbrush Plugs After Five Years, and Relative Similarity of Sites to Rocky Prairie as Determined by Caplow and Chappell[9].

Site	Survival	Flowering	Relative Similarity to Rocky Prairie
Scatter Creek	0.25	0.12	1
Mima Mounds	0.16	0.1	5
West Rocky	0.15	0.06	2
Tenalquot	0.12	0.09	6
Glacial Heritage	0.12	0.09	4
Wolf Haven	0.07	0.07	3

Percentages are calculated based on the total number of plugs outplanted at each site.

**Fig 2:**
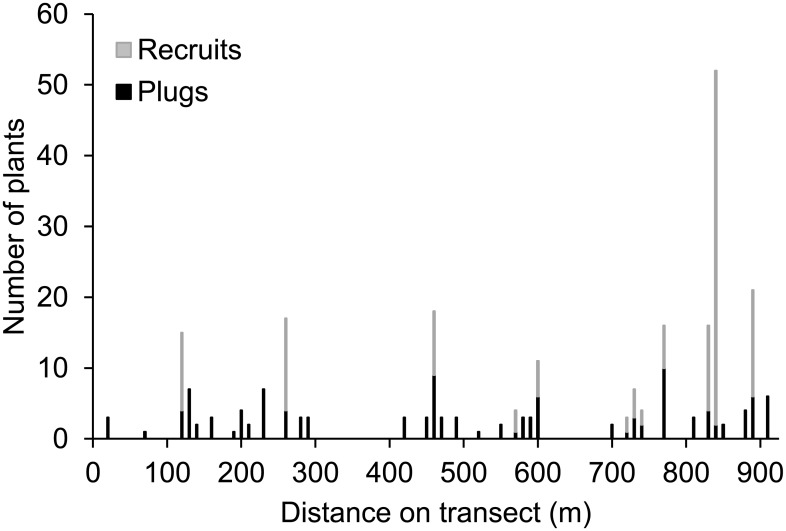
Distribution of golden paintbrush plants along a 920 m transect at Glacial Heritage, five years post-planting. Ten plants were originally planted in groupings at 10 m intervals along the entire transect.

By outplanting golden paintbrush at regular intervals along transects across each prairie, individual plugs ended up in a variety of topographic positions, as well as in association with a variety of different plant species. Thus, it is not surprising that survival and recruitment of golden paintbrush was variable not only between sites, but within sites as well. Both survival and recruitment typically occurred in clusters. For example, Fig 2 illustrates the distribution of golden paintbrush in 2012 along the transect planted at GH in 2007. While all plants died out entirely in many areas, the outplantings survived well in others, and even added many recruits in some areas through reproduction by seed.

A total of 100 species, 60 of them native, were recorded in the quadrats across the six prairies where golden paintbrush was outplanted. Of these, Indicator Species Analysis identified 15 strong positive indicators (IV ≥ 025, *p ≤* 0.05) of golden paintbrush survival. All but three (*Leucanthemum vulgare*, *Plantago lanceolata*, and *Vicia sativa*) were native, and all but two (*Festuca roemeri*, *Carex inops*) were forbs (Table 2). Another 11 species were weak (IV ≤ 24) but significant positive indicators. Nine species were negatively associated with golden paintbrush, and only two of these (*Camassia quamash*, *Lotus micranthus*) were native. In the native golden paintbrush population at Rocky Prairie, 15 species were positive indicators for golden paintbrush; there were no negative indicators.

**Table 2:** Indicator Values of Species Associated with Presence or Absence of Golden Paintbrush.

Species		Site						
	Funct. Group	GH	MM	WH	TQ	SC	WR	RP
*Achillea millifolium*	NPF	15.8	**36.4**	**33.3**	X	**49.3**	X	**41.9**
*Agrostis capillaris*	EPG	**-38.6**	X	**-50.4**	X	X	X	**44.1**
*Aira praecox*	EAG	**-40.2**	-8.3	X	-9.2	X		
*Anthoxanthum odoratum*	EPG	**X**	X	X	X	X	X	**53.3**
*Apocynum androsaemifolium*	NPF			9.8			X	X
*Brodiaea coronaria*	NPF	18.4	X	X	X	X	X	X
*Camassia quamash*	NPF	X	X	**-48.9**	**-50**	X	X	X
*Campanula rotundifolia*	NPF	**54.4**	**43.7**			X	X	
*Carex inops*	NPG	**38.2**	**59.1**	**50.6**	**48.7**	X	X	**53**
*Cytisus scoparius*	ES	X	-8.6	X	X	X	X	X
*Danthonia californica*	NPG	16.3	14.6	7.9	X	X	X	11.4
*Delphnium nuttallii*	NPF						-9.9	X
*Eriophyllum lanatum*	NPF	**53.2**	X	**40.2**	23	X	X	**55.1**
*Festuca roemeri*	NPG	**53.7**	**50.1**	X	X	**51.1**	20.3	**53**
*Fragaria virginiana*	NPF	13.7	**39**	**51.4**	X	X		**46.6**
*Galium aparine*	NAF			10.4	X			X
*Hieracium scouleri*	NPF	12.3	15.4	X	X	X	X	**32.2**
*Hypochaeris radicata*	EPF	**-51.4**	**-49.6**	**-50.3**	X	X	X	**43.1**
*Leucanthemum vulgare*	EPF	**43.8**	**64.2**	**37.7**	X	X	**-49.9**	**29.2**
*Lotus micranthus*	NAF	**-27.5**	-17.2	X	X	X	X	X
*Luzula comosa*	NPG	X	-12	X	X	X	X	X
*Microseris lacinata*	NPF	**31.3**	X	**25.3**	X	X	X	**45.2**
*Myosotis discolor*	EAF			11.8	X			X
*Plantago lanceolata*	EPF	**29.5**	16.9	X	X	X	**-38.8**	X
*Potentilla gracillis*	NPF	16.9	8.9			X		15.4
*Prunella vulgare*	NPF	18.8	X	**31.9**	X	X	X	**39.9**
*Pteridium aquilinum*	FRN	17.6	X	X		**37.7**	20.9	X
*Ranunculus occidentalis*	NPF	**47.1**	**25.2**	**25.1**	X	**43.6**	X	**47.5**
*Rumex acetosella*	EPF	X	X	**-44.1**	X	X	X	X
*Sericocarpus rigidus*	NPF	**23.1**	X	17.1	X	X	X	X
*Solidago missouriensis*	NPF	X		X		X		**39.5**
*Solidago simplex*	NPF	21.9	X		X	16.7		X
*Symphocarpus albus*	NS			13.7		X	X	X
*Teesdalia nudicaulis*	EAF	**-46.8**	**-46**	**-49.9**	X	X	X	**41.3**
*Trifolium pratense*	EPF		5.7					
*Veronica arvense*	EAF	X	X	21.6	-8.4	-11.9	X	
*Vicia sativa*	EAF	12.4	X	X	**40.3**	X	X	X
*Viola adunca*	NPF	**45.5**	**52.8**	**47.1**		X	X	15.8
*Zigadenus venenosus*	NPF	16.2	X	X	X			11.1

GH, MM, WH, TQ, SC, WR are outplanted sites (full site names are listed in text). RP is a natural golden paintbrush population. Significant Indicator Values (IV ≥ 25, p<0.05) are **bolded**, small IV values (<25) that are statistically significant are unbolded. “X”, species present in quadrats but not statistically significant; blank, species not present in any quadrats at a site. Functional groups: N, Native; E, Exotic; P, Perennial; A, Annual; F, Forb; G, Graminoid; S, Shrub. Note that several species occur as both positive and negative indicators at different sites.

When looking at the associated functional groups instead of by species, perennial forbs (both native and exotic) and native perennial grasses were positively associated (*p*<0.05) with golden paintbrush presence (Table 3). Significant negative indicators included annual forbs (native and exotic) and exotic annual grasses.

**Table 3:** Indicator Values of Golden Paintbrush by Plant Functional Group.

Functional group	Golden	Specificity (Mean A)	Frequency (Mean B)	Mean IV	P-value
Native Perennial Forbs		0.56	1	55.91	0.000*
Native Perennial Grasses		0.55	0.99	54.44	0.000*
Exotic Perennial Forbs		0.53	0.99	52.4	0.007*
Native Shrubs		0.81	0.09	7.02	0.069
Native Annual Forbs		0.67	0.32	-20.34	0.006*
Exotic Annual Forbs		0.55	0.79	-42.2	0.009*
Exotic Annual Grasses		0.73	0.23	-15.61	0.027*
Exotic Perennial Grasses		0.51	0.97	-49.36	0.085

Indicator Values by functional group (across all six outplanting sites). Specificity (Mean A) describes how often a functional group is associated with a given variable (1 = always found with golden paintbrush). Frequency (Mean B) is the occurrence of a group across all plots (1 = found in every plot). P-values <0.05 indicated with asterisk (*).

**Fig 3:**
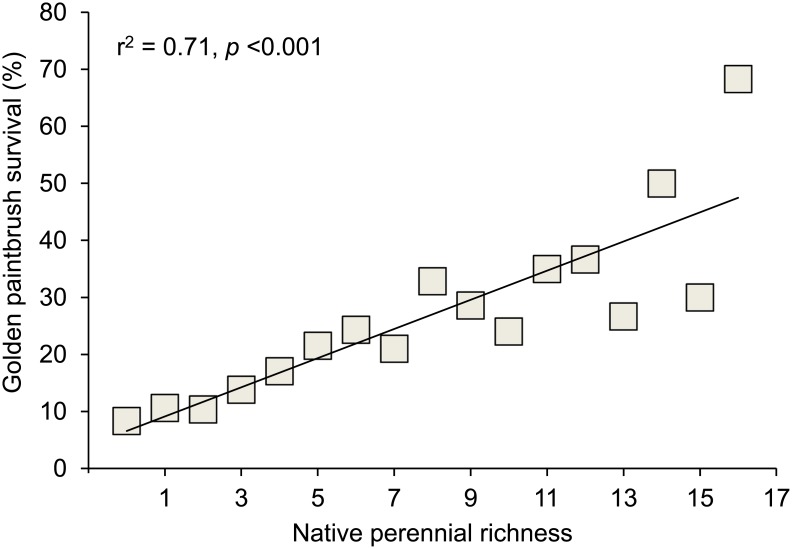
Native perennial richness versus survival of golden paintbrush.

When averaged across all six sites, golden paintbrush survival was strongly correlated with native perennial richness (r^2^ = 0.71, *p*<0.001; Fig 3). A similar but slightly weaker relationship was found between native perennial richness and the proportion of quadrats that contained flowering golden paintbrush (r^2^ = 0.55, *p*<0.001).

**Fig 4:**
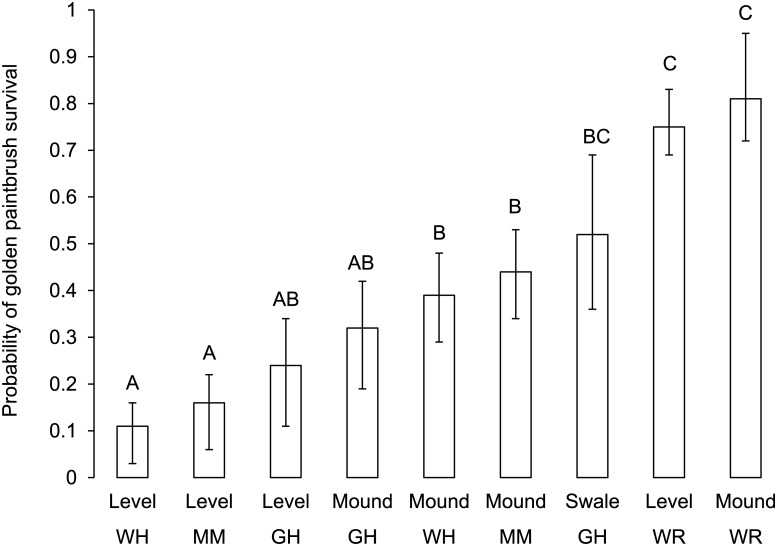
The influence of prairie and topography on the probability of golden paintbrush survival after 5 years at four prairies with topographic heterogeneity (GH = Glacial Heritage, MM = Mima Mounds, WH = Wolf Haven, WR = West Rocky). Error bars represent 95% confidence intervals. Different letters represent significantly different probabilities of survival (α ≤ 0.05).

Topography and soils, which are closely linked in these prairies, also affected golden paintbrush survival, though this influence depended upon site (Fig 4). Both mounds and swales are generally where the deepest soils occur in gravelly prairies, and typically are composed of Nisqually soils. Such topography exists at four of the sites (MM, GH, WR, WH). At the two most heavily mounded prairies (MM and WH), paintbrush survival was much higher on mounds (MM = 46% higher and WH = 56%) than in the level interspaces. While mounds were also present at GH, survival was only 14% greater on them, compared to level habitat, and swales had the strongest positive influence on survival (37% higher than level habitat, 24% greater than mounds). Paintbrush survival was highest at WR compared to the three other mounded sites regardless of topographic position. The lack of topographic influence at WR is likely due to the differences in soils, which are primarily Nisqually throughout.

**Fig 5:**
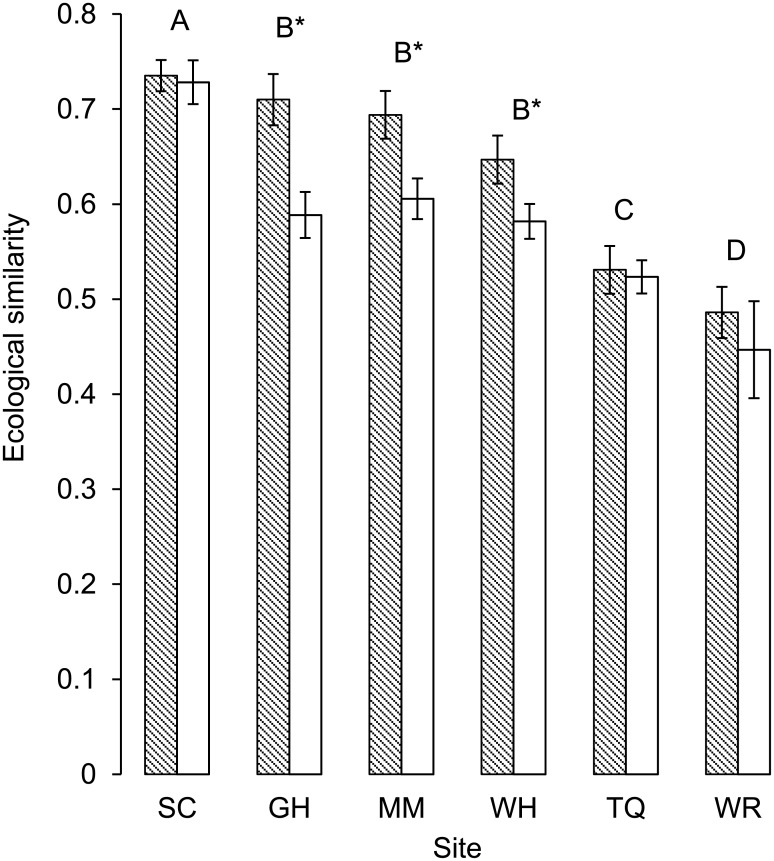
Ecological similarity of microsites within six reintroduction sites compared to microsites at Rocky Prairie. Different letters denote significant difference across sites (α = 0.05), “*” denote prairies where ecological similarity was significantly different between where golden paintbrush was absent or present (α = 0.05).

Ecological similarity with Rocky Prairie microsites was significantly different across outplanted sites; SC was most similar, while TQ and WR were the least. At the microsite level, ecological similarity was only important in distinguishing the microsites where golden paintbrush survived at three sites (GH, MM, and WH) (Fig 5). However, similarity at the site level did not correlate well with overall survival at each site (r^2^ = 0.17, *p* = 0.2231).

## Discussion

There are significant incentives to reduce costs and improve the efficiency of rare species restoration efforts. Over the past 15 years, substantial resources have been directed towards augmenting existing populations and establishing new populations of golden paintbrush in Washington, Oregon, and British Columbia. Until 2011, these efforts involved extensive propagation and outplanting of nursery-grown plugs. Outplanting plugs is frequently recommended in rare species recovery efforts over directly sowing seed in the field [4] due to the much higher establishment rate typical of most plantings. However, there are significant tradeoffs with these approaches that can make choosing between them less clear cut. Producing plugs can be expensive; currently, golden paintbrush plugs produced by Center for Natural Lands Management (CNLM) nurseries cost about $3.00 each compared with nursery-grown seed at ca. $.30 per 1000 seeds. Furthermore, outplanting costs are much higher than sowing seed. These differences in cost must be balanced against differences in survival. We have found that on suitable sites an average of one-third of outplanted plugs survive two years; in contrast, seed germination and survival in the field is typically low, generally <1% (Dunwiddie, unpublished data). However, survival of both plugs and seed varies tremendously, even within generally suitable sites (Table 1, Fig 2). Since 2011, as large quantities of golden paintbrush seed have become available from nursery production beds, we have shifted reintroduction efforts away from outplanting plugs at many sites, and begun to rely largely on the direct sowing of seed, which overall is considerably more cost-effective. This approach also somewhat reduces the necessity of precisely identifying suitable microsites; if large enough areas can be seeded, at least some seed is likely to fall in more optimal locations. However, identifying indicators of the most suitable locations within sites can greatly increase overall survival, reduce costs, and maximize reintroduction effectiveness.

Typically, sites for establishing new populations of rare species are selected by identifying key characteristics (e.g., topography, soils, vegetation) that describe sites where the species is extant, and evaluating how the same characteristics at potential sites compare with the reference sites [7]. This approach has been used with golden paintbrush as well, drawing on comparisons with characteristics of all extant sites [9,22,32]. Caplow and Chappell [9] conducted a detailed ranking of potential sites in South Puget Sound by comparing them with the single extant site in the region (Rocky Prairie). Table 1 compares their rankings with how the 2007 golden paintbrush outplantings performed at these sites. This comparison illustrates problems with both approaches to evaluating potential site suitability. A major weakness of the approach used by Caplow and Chappell is that it assumes reference sites (or in this case, a single site) represent conditions most suitable for survival of the species. However, Falk et al. [7] and others have noted rare species often have been eliminated from many of the most suitable sites, and may be persisting only in a few, relatively marginal sites. We strongly suspect this to be the case with golden paintbrush, as recent studies by Delvin [23] have demonstrated that extremely large populations (>100,000 plants) of golden paintbrush can be established in prairies restored in former agricultural lands, which tend to have deeper soils than most existing native prairie remnants. Although they generally retain no native taxa, they may be prime golden paintbrush sites when restored. Thus, site-selection approaches that include criteria based on extant native vegetation are likely to reject some sites that may otherwise be optimal, but where native vegetation may be entirely lacking.

Outplanting of rare species in sites using a more rigorous, experimental approach, such as recommended by Guerrant and Kaye [8], provides more directly measurable evidence of site suitability. One such study has been carried out with golden paintbrush in Oregon, where Lawrence and Kaye [32] conducted a common garden study to examine factors contributing to reintroduction performance and survival. When such outplantings are carried out systematically across multiple sites, they may provide a means for ranking their relative suitability (Table 1). However, there are limitations to this approach as well. First, such plantings are costly, and should only be undertaken where there is reasonable evidence that they will be at least modestly successful, such as provided by the assessments carried out at our sites by Caplow and Chappell [9]. Second, sites that have variable soil and vegetation conditions may require extensive outplantings to sufficiently evaluate the entire range of conditions. We used large numbers of plants that were well dispersed across each site to help capture the within-site variability. Third, some sites may provide highly suitable habitat for a rare species if managed appropriately, but may have existing conditions rendering them less suitable. If such conditions are not recognized and corrected before the plants are installed, plants may perform relatively poorly, and the sites may therefore appear to be relatively unsuitable. Wherever possible at our sites, we located the outplanting transects to include some areas that had been recently burned, where significant control of invasive species had been undertaken, and where some native species had been seeded—all treatments we believed would enhance conditions favorable to growth and establishment of golden paintbrush.

Two examples from our sites illustrate these complexities. WH ranked last or next-to-last for survival and flowering in our study, and was deemed only moderately suitable by Caplow and Chappell [9]. However, subsequent management of the site by burning has reduced deep accumulations of moss and thatch, invasive weeds have been controlled using herbicides, and native species diversity has been increased by seeding. These actions have greatly enhanced golden paintbrush establishment and survival. Although the initial plantings in 2007 did relatively poorly, subsequent seedings have built the population to now exceed 3600 flowering plants. The GH site highlights different sorts of issues. Based on their assessment of the 490 ha native prairie on the site, Caplow and Chappell [9] ranked it fourth out of the six South Sound prairies. Our plantings, also carried out in the same area, yielded a similar ranking. However, in a small and largely overlooked 3 ha fragment of Nisqually soils that was entirely dominated by exotic pasture grasses, aggressive management restored native prairie vegetation, and a population of golden paintbrush in excess of 100,000 plants has been established [23].

Even when a site may be considered generally suitable for sustaining a population of a rare species, targeting plantings towards specific areas representing the most suitable conditions can greatly reduce costs and increase success. The patchy survival and recruitment of golden paintbrush along the transect at GH (Fig 2) was typical of the other study sites as well, and suggests that even within a site, suitability must be assessed at several scales. These results confirmed our impression that golden paintbrush habitat suitability varied considerably within sites, and the recognition and exclusion of unsuitable microsites could lead to considerable savings in plugs or seeds. For example, survival at GH was 12%. However, it varied from 9% in the least suitable microsites (level prairie with ≤ 4 native perennial species) to 36% in the most suitable microsites (mounds or swales with ≥7 native perennial species), a four-fold increase.

Our analyses identified several useful characteristics that may indicate the most suitable microsites for planting golden paintbrush. The presence of particular species provides some indication of potential success. Microsites with abundant native species such as *Festuca roemeri*, *Eriophyllum lanatum*, *Ranunculus occidentalis*, *Viola adunca*, *Carex inops*, and *Achillea millefolium* are likely to be particularly promising, whereas microsites with extensive non-natives, such as *Hypochaeris radicata*, *Agrostis stolonifera*, and *Teesdalia nudicaulis* are less likely to be suitable. Although none of the individual species that were positive indicators were strongly associated with golden paintbrush survival at every site, our results showed a strong positive correlation with the total number of native perennials. Many species were not strong indicators at some sites simply because they occurred at low frequencies, which precluded calculation of meaningful values of statistical significance. Further, species not being significant indicators at all sites is expected because other variables, such as soil conditions or species composition, can override strong species relationships, a pattern that has been reported with other hemiparasites [33]. Therefore, finding microsites with a high diversity of native perennials, particularly when they include several of the positive indicator species, may be important for identifying especially suitable sites for golden paintbrush planting. This also suggests that creating “hotspots” of diverse perennial forbs when restoring prairies may assist in increasing their suitability for golden paintbrush recovery.

The mechanisms underlying these relationships are uncertain. Multiple factors may be influencing outcomes, either individually or in combination. Positive indicator species could be associated with golden paintbrush because they serve as important host plants. Three of the fifteen positive indicator species are confirmed hosts (*Achillea millefolium*, *Eriophyllum lanatum*, *Festuca roemeri* [23]), and others are likely as well. Alternatively, positive indicator species may simply reflect the presence of abiotic conditions, such as deeper, more nutrient-rich soils, that may favor them as well as golden paintbrush. We suspect many of the species with particularly high IV scores at multiple sites (mostly perennial forbs and grasses) are likely host plants, whereas those with low but statistically significant IV’s share preferences for similar abiotic conditions, but are not important hosts for golden paintbrush. In contrast, many of the negative indicator species were annuals or had root morphologies (bulbs, taproots) that may be less receptive to haustorial connections with hemiparasites.

The strong positive correlation with high species richness also may reflect multiple mechanisms. First, since generalist hemiparasites can parasitize multiple hosts simultaneously [34], golden paintbrush may be more successful where a combination of hosts are available [20], and the paintbrush may benefit from several potentially complementary resources [35]. Second, higher plant diversity at a microsite may simply increase the chance that a particularly suitable host is available, thereby increasing the likelihood of golden paintbrush surviving. Third, abiotic conditions favoring golden paintbrush may also sustain a larger number of other species than are able to thrive in other microsites. Although we did not measure soil depth and texture directly, the positive association of golden paintbrush with mounds and swales suggests an affinity for deeper, less-rocky soils that may be more productive or have higher moisture retention. Yet a fourth possibility is that parasitism by golden paintbrush on various species may be helping to enhance species diversity, a pattern that has been suggested with other hemiparasites [36]. Understanding which of these mechanisms are the strongest determinants of the patterns we observed will require more investigation.

Comparisons with microsites where golden paintbrush grows naturally at Rocky Prairie help to explain some of the complexities in our results. In the ISA analysis, all of the species that were strong indicators at any of the six outplanting sites were also strong indicators at Rocky Prairie, when those species were present there (Table 2). However, other species at Rocky Prairie were unexpectedly strong indicators, such as the weedy annual *Teesdalia nudicaulis*. Even more surprising was the lack of any species being negative indicators at this site. These results may support Hartley et al.’s [36] suggestion that the interactions of hemiparasites with the surrounding community enhances species diversity. These effects may be most evident in a site where golden paintbrush has been well established for (presumably) centuries, and may become increasingly evident over time at our experimental sites as the competitive interactions brought about by its parasitism on other species help to shape the overall community composition.

Identifying sites that are most ecologically similar to reference sites is frequently mentioned as an important element in selecting the most suitable sites for reintroducing rare species [37]. The common garden study using golden paintbrush in Oregon by Lawrence and Kaye [32] found that ecological similarity with functional groups at reference sites was related to short-term survival of outplanted golden paintbrush. However, our analyses of sites in South Puget Sound in Washington suggest using ecological similarity of vegetation alone at the site level may have little utility in predicting longer term site suitability for golden paintbrush. Nor did the site comparisons made by Caplow and Chappell [9] using soils, topography, and a coarser, site-level measure of vegetation composition, predict golden paintbrush survival in our plantings (Table 1 and Fig 4).

Rocky Prairie has a site history that is unlike other extant prairie remnants in the region. The site has relatively high native species richness and low exotic species richness. At Rocky Prairie, 89% of quadrats with golden paintbrush have more than 7 native perennials, compared to only 22% of the quadrats across all the reintroduction sites. Unlike these other sites, Rocky Prairie has no history of grazing or intensive human alteration; hence, it presents conditions not currently found in the six reintroduction sites used in this study, or in any of the remaining prairie habitats in the South Puget Sound region. Efforts are being made to increase native diversity at some of these sites, but soil differences may preclude restoring most of them to closely resemble Rocky Prairie.

While ecological similarity to Rocky Prairie varied among the reintroduction sites (Fig 4), the relationship to outplanted golden paintbrush survival was complex. In microsites where outplanted paintbrush survived, ecological similarity was greater than where paintbrush failed to survive at only three of the six sites (GH, MM, WH). Furthermore, the most dissimilar site (WR) had the third highest survival. These complexities make it difficult to generalize conclusions regarding ecological similarity measures. SC had the greatest ecological similarity to Rocky Prairie (Fig 4), but this measure failed to distinguish those microsites where paintbrush survived. SC is a flat, relatively uniform site, and like Rocky Prairie, is dominated by Nisqually soils and has relatively high native perennial richness (58% of quadrats had > 7 native perennial species). Thus, differences in survival among the microsites apparently were driven by unmeasured factors, since both soils and vegetation were similar to Rocky Prairie. Conversely, at the two sites most dissimilar to Rocky Prairie (TQ and WR), microsites had low ecological similarity (both TQ and WR had only 3% of quadrats with more than 7 native perennial species), and this measure had no detectable influence on paintbrush survival. As expected, golden paintbrush survival was poor at TQ because the site has very low native perennial richness and no Nisqually soils. At WR, similarity in soils to Rocky Prairie (both were historically part of the same prairie) rather than similarity in vegetation may have driven the higher rates of plug survival. Ecological similarity was most important in predicting golden paintbrush survival at sites where microsite conditions were most variable—in the mounded and swale topography at GH, MM, and WH.

These results suggest that microsites with species-rich native vegetation assemblages may not be as important for golden paintbrush survival in Nisqually soils, where this species typically may have occurred historically. However, few prairie remnants exist today on this soil type; most were long ago converted to agriculture. As a result, golden paintbrush restoration efforts instead have been directed towards the few remaining prairie fragments, which largely exist on Spanaway or Spanaway-Nisqually complex soils. These appear to be sub-optimal locations for golden paintbrush recovery except in microsites where native richness is high, host species are present, and soils more closely resemble Nisqually conditions. Microsite selection, therefore, is particularly important at these sites with mixed soils, varied topography, and heterogeneous plant communities. Managing these sites for high richness of native species with a multitude of hosts should be a priority where golden paintbrush restoration is a goal. Furthermore, reintroduction of paintbrush may also be successful on sites with Nisqually or similarly productive soils. Although such restoration likely will involve converting agricultural fields back into a native prairie assemblage, our results suggest establishing a diverse native assemblage on these soils may be less critical for golden paintbrush survival. Recent efforts to establish new golden paintbrush populations in two former agricultural fields on Nisqually soils suggest this approach may be very successful [23].

## Conclusions

The cost and success of establishing new populations of rare species are strongly dependent on ensuring that outplantings are carried out in locations favorable for establishment, growth, survival, and reproduction. We found that even in sites considered to be generally suitable, survival rates of outplanted plugs often varied widely across the site. By examining floristic and abiotic characteristics of microsites where survival was highest, we identified attributes that practitioners can use to select locations where future golden paintbrush plantings are likely to be most successful. We suggest that recovery efforts for other rare plants may be more effective and efficient if experimental outplantings are designed and carried out to identify specific characteristics of highly suitable habitats at small spatial scales across multiple sites. Furthermore, the success of plantings will increase with focused efforts to exclude sub-optimal microsites.

## Supporting Information

S1 Table Data used in analyses. (XLSX)
